# Comparative diagnostic accuracy of different artificial intelligence models for early gastric cancer: a systematic review and meta-analysis

**DOI:** 10.3389/fonc.2025.1670843

**Published:** 2025-11-18

**Authors:** Minfang Lv, Fei Chen, Qinghai Li, Meng Xue, Jun Wang

**Affiliations:** 1 Nursing Department, The Second Affiliated Hospital, Zhejiang University School of Medicine, Hangzhou, Zhejiang, China; 2 Department of Gastroenterology, The Second Affiliated Hospital, Zhejiang University School of Medicine, Hangzhou, Zhejiang, China; 3 Department of Radiology, The Second Affiliated Hospital, Zhejiang University School of Medicine, Hangzhou, Zhejiang, China; 4 Department of Gastroenterology Surgery, The Second Affiliated Hospital, Zhejiang University School of Medicine, Hangzhou, Zhejiang, China

**Keywords:** artificial intelligence, early gastric cancer, endoscopy, diagnosis, meta-analysis

## Abstract

**Objective:**

Timely diagnosis of early gastric cancer (EGC) is significantly associated with patient prognosis, but traditional endoscopic diagnosis relies on the physician’s experience and has certain limitations. This study comprehensively evaluated the accuracy of artificial intelligence (AI) in the diagnosis of EGC through meta-analysis and compared the performance ability of different AI models.

**Methods:**

PubMed, Embase, Web of Science Cochrane Library, and China National Knowledge Infrastructure databases were systematically searched (established until January 2025), and studies evaluating the accuracy of AI models in the diagnosis of EGC were included, requiring reporting of sensitivity and specificity, or providing data for calculating these indicators. Data were extracted independently by two reviewers, and sensitivity and specificity were pooled using a bivariate random effects model, and subgroup analysis was performed by AI model type. The primary outcome measures were the summary sensitivity, specificity, and area under the curve (AUC) of all AI models.

**Results:**

Of 26 studies involving 43,088 patients were included. Meta-analysis results showed that the summary sensitivity of the AI model was 0.90 (95%CI: 0.87-0.93), the specificity was 0.92 (95%CI: 0.87-0.95), and the AUC was 0.96 (95%CI: 0.94-0.98), respectively. Subgroup analysis showed that the sensitivity of deep convolutional neural network (DCNN) was higher than that of traditional CNN (0.94 vs 0.89), while the specificity was almost equivalent (0.91 vs 0.91). In dynamic video verification, the AUC of the AI model reached 0.98, which was significantly better than the clinician level (AUC 0.85-0.90).

**Conclusion:**

The AI model, especially the DCNN architecture, showed excellent accuracy in the diagnosis of EGC. Future research should focus on the dynamic effect of the model, improvement of interpretability, and multicenter prospective validation.

**Systematic Review Registration:**

https://www.crd.york.ac.uk/PROSPERO/view/CRD420251003071, identifier CRD420251003071.

## Introduction

Gastric cancer is one of the malignant tumors with high morbidity and mortality worldwide (1). Its early diagnosis is crucial to improving the prognosis of patients. Early gastric cancer (EGC) refers to cancer confined to the gastric mucosa or submucosal layer (2). If diagnosed in time and treated with minimally invasive treatments such as endoscopic submucosal dissection (ESD), the 5-year survival rate of patients can exceed 70%, and the medical burden is also lower than that of advanced gastric cancer (3, 4). However, the endoscopic diagnosis of EGC faces huge challenges: its lesions often show subtle color changes on the mucosal surface, abnormal microvascular structure or mild protrusion/depression (5, 6). These morphological features are easily overlooked, especially in primary medical institutions or among junior endoscopists, and the misdiagnosis rate can over 20% (7, 8).

In recent years, artificial intelligence (AI) technology has demonstrated significant advantages in the field of endoscopic image analysis through breakthroughs in deep learning (DL) and generic convolutional neural networks (CNN) (9, 10). Studies have shown that AI can automatically extract the texture, morphology and microvascular pattern features of lesions to achieve accurate identification of EGC (9, 10). For example, the EfficientNetB7 model has an accuracy rate of 97.88% in diagnosing early gastric cancer in white light endoscopic images, significantly exceeding the level of traditional physicians (11). In contrast, a 2021 systematic review and meta-analysis by Jiang et al. (12), analyzed 16 studies and found that AI-assisted endoscopic detection of EGC achieved a pooled sensitivity of 0.86, specificity of 0.93, and an area under curve (AUC) of 0.96. But its data only covered August 2022, and did not systematically compare the differences between different model architectures.

With the rapid iteration of AI technology, new algorithms such as deep convolutional neural networks (DCNN), an advanced CNN variant with deeper layers for hierarchical feature extraction, unlike generic CNNs with shallower architectures, and hybrid architecture models (such as the HistoCell algorithm) continue to emerge (13). DCNN differ from generic CNN by incorporating residual connections and batch normalization, enabling better gradient flow and performance on complex endoscopic images. Their ability to analyze the association between pathological images and molecular networks at the single-cell scale provides new ideas for the very early warning of EGC (13). At the same time, the application of dynamic endoscopic video analysis technology has increased the detection rate of EGC through real-time quality control and blind spot monitoring, and significantly improved the number of biopsies (14–16). However, there are few major limitations in existing studies: First, most evidence is based on retrospective static images and lacks prospective video verification; Second, the performance differences of different models (CNN, DCNN, SVM) have not been quantified; Third, the sources of publication bias and heterogeneity (such as endoscopic equipment type, imaging technology) have not been fully explored.

Based on this, this study aims to comprehensively evaluate the effectiveness of AI in the diagnosis of EGC through systematic review and meta-analysis, quantify the diagnostic differences among CNN, and non-DL models (such as SVM) through subgroup analysis, and explore the application prospects of AI in the diagnosis of early gastric cancer.

## Methods

This study followed the Preferred Reporting Items for Systematic Reviews and Meta-Analyses (PRISMA) guidelines and was registered with PROSPERO (registration number: CRD420251003071).

### Search strategy

We searched the following databases: PubMed, Embase, Web of Science, Cochrane Library, and China National Knowledge Infrastructure (CNKI) from inception to January 31, 2025. We used the following keywords and their MeSH word combinations: “artificial intelligence”, “machine learning”, “deep learning”, “convolutional neural network”, “support vector machine”, “random forest”, “early gastric cancer”, “endoscopy”, “diagnosis”, and “accuracy”. The specific search formula is shown in **Supplementary Table 1**. The search language was limited to Chinese or English, and the references of the included studies and related reviews were manually checked to supplement the missing studies.

### Inclusion and exclusion criteria

#### Inclusion criteria

The study aims to evaluate the performance of AI models in the diagnosis of EGC, using endoscopic images or videos as input data. Provide sensitivity, specificity, or raw data that can calculate these indicators (such as true positive, false positive, true negative, false negative counts). Histopathological examination is used as the gold standard for the diagnosis of EGC. The study was published in a peer-reviewed journal and the full text is available.

#### Exclusion criteria

The type of AI model was not clearly stated or the diagnostic performance indicators were not reported. The subjects were advanced gastric cancer or other gastrointestinal diseases. Non-original research (such as case reports, reviews, conference abstracts).

### Data extraction

Two reviewers independently extracted data using a pre-designed form. Data on study characteristics included authors, year of publication, country, study design (prospective or retrospective), and sample size. Patient characteristics included the number of EGC cases and endoscope type (white light endoscopy, narrow band imaging (NBI), etc.). For NBI, it was defined as “traditional NBI, with or without magnification as per the primary study protocol. AI model characteristics included model type (CNN, SVM, RF, etc.) and training dataset size. Diagnostic performance data included sensitivity, specificity, true positive (TP), false positive (FP), true negative (TN), false negative (FN), and area under curve (AUC). If data were missing, the author was contacted for supplementary information. If there were any disagreements during the extraction process, a third reviewer assisted in resolving them.

### Quality assessment

The risk of bias and applicability of included studies were assessed using the Quality Assessment Tool for Diagnostic Accuracy Studies (QUADAS-2) (17). The risk of bias assessment mainly covers four areas. In terms of patient selection, it is determined whether patients are included continuously and whether there is selection bias. In terms of index testing, it is evaluated whether the implementation and validation of the AI model are clearly described. In terms of reference standards, it is determined whether all patients undergo histopathological examination. In terms of process and time, it is determined whether the time interval between the test and the reference standard is reasonable. The risk of each study is divided into “low”, “high” or “unclear”.

### Statistical analysis

The pooled analysis was performed using the Meta Disc 2.0 tool (18, 19). For sensitivity and specificity, 95% confidence intervals (CI) were calculated, and summary receiver operating characteristic curves (SROC) were drawn, and AUC was reported. Subgroup analysis was divided into CNN or non-CNN according to the type of AI model to explore the impact of model architecture on diagnostic performance.

Heterogeneity was assessed using the I² statistic and the χ² test. If I²>50%, significant heterogeneity was considered, and the sources (such as study design, sample size, endoscope type, model type) were explored by subgroup analysis or meta-regression analysis if possible. Publication bias was assessed using funnel plots and Egger’s test, and p<0.05 was considered significant (20). All analyses were completed using R software (version 4.4.3) and its “mada” and “meta” packages. The impact of individual studies on the overall results was assessed by excluding each study one by one to ensure the robustness of the results. The results were considered statistically significant at p<0.05.

## Results

General information and baseline characteristics of the included studies

**Figure 1:**
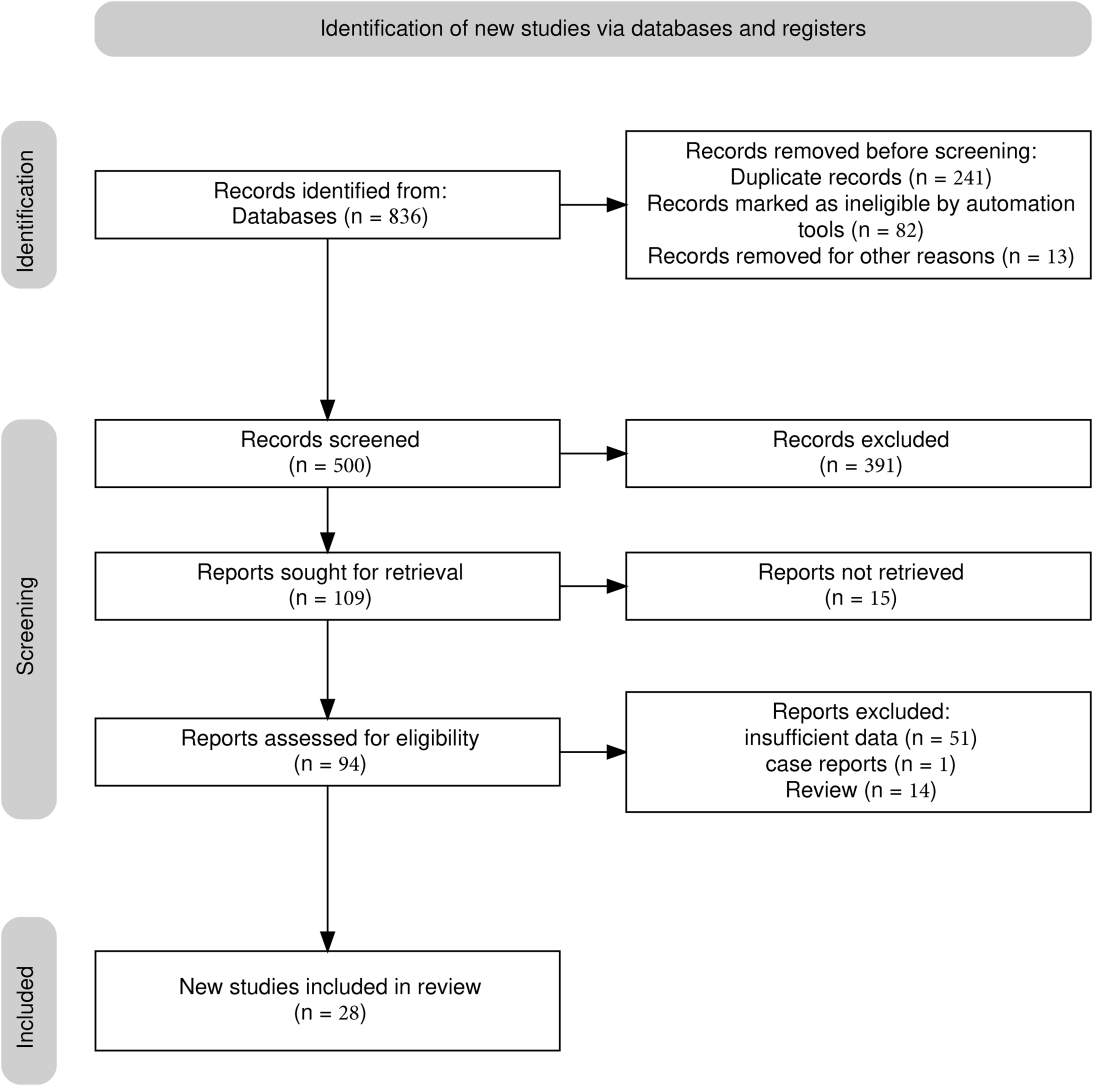
PRISMA flowchart.

**Figure 2:**
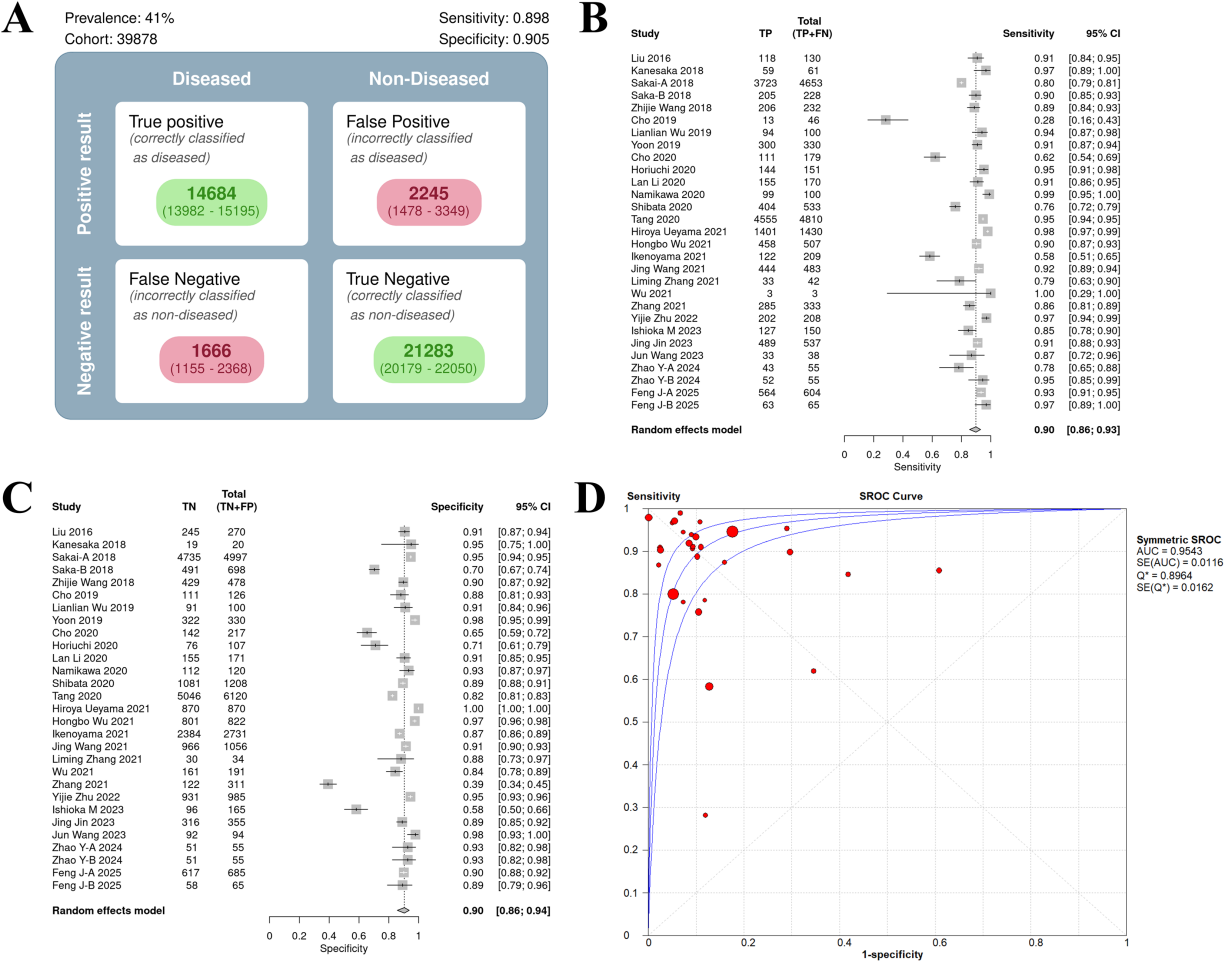
Univariate model analysis results of AI model diagnostic performance. **(A)** summary result of pooled data. **(B)** Forest plot of sensitivity. **(C)** Forest plot of specificity. **(D)** SROC curve.

A total of 26 studies (21–46) were included (**Figure 1**), with a total of 39878 cases, including 18097 EGC cases (45.38%) and 21781 non-EGC cases (54.62%), covering endoscopic images and video data (**Figure 2A**). The publication years spanned from 2016 to 2025. Among them, the most studies were published in 2021, followed by 2020 and 2023. The studies mainly came from China and Japan (**Table 1**).

**Table 1:** Baseline characteristics of the included studies.

Study	AI model	Country	Prospective/retrospective	Imaging	Label	EGC/total
Wu 2021	CNN	China	Prospective	WLI	CNN	3/194
Cho 2019	CNN	Korea	Prospective	WLI	CNN	46/172
Jun Wang 2023	GAIDS	China	Retrospective	NBI	GAIDS	38/160
Liming Zhang 2021	CNN	China	Retrospective	WLI	CNN	42/76
Kanesaka 2018	SVM	Japan	Retrospective	NBI	SVM	61/81
Lianlian Wu 2019	DCNN	China	Retrospective	WLI_NBI	DCNN	100/200
Namikawa 2020	CNN	Japan	Retrospective	WLI_NBI	CNN	100/220
Cho 2020	CNN	Korea	Retrospective	WLI	CNN	179/396
Liu 2016	SVM	China	Retrospective	WLI	SVM	130/400
Ikenoyama 2021	CNN	Japan	Retrospective	WLI_NBI	CNN	209/2940
Ishioka M 2023	CNN	Japan	Retrospective	WLI	CNN	150/315
Horiuchi 2020	CNN	Japan	Retrospective	NBI	CNN	151/258
Lan Li 2020	CNN	China	Retrospective	NBI	CNN	170/341
Yijie Zhu 2022	DCNN	China	Retrospective	NBI	DCNN	208/1193
Zhijie Wang 2018	DCNN	China	Retrospective	WLI	DCNN	232/710
Zhang 2021	CNN	China	Retrospective	WLI	CNN	333/644
Yoon 2019	CNN	Korea	Retrospective	WLI	CNN	330/660
Shibata 2020	CNN	Japan	Retrospective	WLI	CNN	533/1741
Jing Wang 2021	DCNN	China	Retrospective	WLI	DCNN	483/1539
Hongbo Wu 2021	CNN	China	Retrospective	WLI_Video	CNN	507/1329
Jing Jin 2023	CNN	China	Retrospective	WLI_NBI	CNN	534/892
Hiroya Ueyama 2021	CNN	Japan	Retrospective	NBI	CNN	1430/2300
Sakai 2018	CNN	Japan	Retrospective	WLI	CNN	4653/9650
Tang 2020	CNN	China	Retrospective	WLI	CNN	4810/10930
Feng J 2025	DCNN	China	Prospective	WLI_NBI_Video	DCNN	604/1289
Zhao Y 2024	CNN	China	Retrospective	WLI_LCI	CNN	55/110

EGC, early gastric cancer; CNN, Convolutional Neural Network; GAIDS, Gastrointestinal Artificial Intelligence Diagnostic System; SVM, Support Vector Machine; DCNN, Deep Convolutional Neural Network; WLI, White light imaging; NBI, Narrow Band Imaging; LCI, Linked Color Imaging.

Among the included studies, 23 retrospective studies accounted for approximately 88.46%, while 3 studies (25, 36, 46) were prospective (11.54%). Regarding the types of AI models, 21 studies (80.77%) utilized CNN, which included classic CNN and their improved architectures. There were 2 SVM (7.69%) studies (21, 22). Additionally, 1 study (44) used other models, but no details about this model was given. For endoscopic imaging technology, 18 studies (69.23%) applied non - narrow - band imaging (Non - NBI), which might be considered as white light endoscopy (WLI), combined imaging (WLI + NBI), linked imaging (LCI), or video dynamic analysis as indicated in the original text. Seven studies (26.92%) used narrow band imaging (NBI).

### QUADAS-2 quality assessment

All studies used histopathology to ensure the reliability of diagnostic accuracy assessment (**Table 2**). They covered a variety of endoscopic imaging techniques (WLI, NBI, or LCI) and AI models (CNN, DCNN, or SVM), reflecting current research trends. The methodological quality of the 26 studies included in this meta-analysis was evaluated using the QUADAS-2 tool, which assesses both risk of bias and applicability concerns across four domains: patient selection, index test, reference standard, and flow and timing. The results provide insight into the reliability and generalizability of the findings.

**Table 2:** QUADAS-2 quality assessment results of included studies.

Study	Risk of bias				Applicability concerns		
	Patient selection	Index test	Reference standard	Flow and timing	Patient selection	Index test	Reference standard
Wu 2021	Low Risk	Low Risk	Low Risk	Low Risk	Low Risk	Low Risk	Low Risk
Cho 2019	High Risk	High Risk	Low Risk	Unclear Risk	High Risk	High Risk	Low Risk
Jun Wang 2023	Low Risk	Low Risk	Low Risk	Low Risk	Low Risk	Low Risk	Low Risk
Liming Zhang 2021	Low Risk	Low Risk	Low Risk	Low Risk	Low Risk	Low Risk	Low Risk
Zhao Y 2024	High Risk	High Risk	Low Risk	Low Risk	High Risk	High Risk	Low Risk
Kanesaka 2018	Low Risk	Low Risk	Low Risk	Low Risk	Low Risk	Low Risk	Low Risk
Feng J 2025	High Risk	High Risk	Low Risk	Unclear Risk	High Risk	High Risk	Low Risk
Lianlian Wu 2019	Low Risk	Low Risk	Low Risk	Low Risk	Low Risk	Low Risk	Low Risk
Namikawa 2020	Low Risk	Low Risk	Low Risk	Low Risk	Low Risk	Low Risk	Low Risk
Cho 2020	Low Risk	Low Risk	Low Risk	Low Risk	Low Risk	Low Risk	Low Risk
Liu 2016	Low Risk	Low Risk	Low Risk	Low Risk	Low Risk	Low Risk	Low Risk
Ikenoyama 2021	Low Risk	Low Risk	Low Risk	Low Risk	Low Risk	Low Risk	Low Risk
Ishioka M 2023	Low Risk	Low Risk	Low Risk	Low Risk	Low Risk	Low Risk	Low Risk
Horiuchi 2020	High Risk	High Risk	Low Risk	Low Risk	High Risk	High Risk	Low Risk
Lan Li 2020	High Risk	High Risk	Low Risk	Low Risk	High Risk	High Risk	Low Risk
Yijie Zhu 2022	Low Risk	Low Risk	Low Risk	Low Risk	Low Risk	Low Risk	Low Risk
Zhijie Wang 2018	Low Risk	Low Risk	Low Risk	Low Risk	Low Risk	Low Risk	Low Risk
Zhang 2020	Low Risk	Low Risk	Low Risk	Low Risk	Low Risk	Low Risk	Low Risk
Yoon 2019	Low Risk	High Risk	Low Risk	Low Risk	Low Risk	High Risk	Low Risk
Shibata 2020	High Risk	High Risk	Low Risk	Low Risk	High Risk	High Risk	Low Risk
Jing Wang 2021	Low Risk	Low Risk	Low Risk	Low Risk	Low Risk	Low Risk	Low Risk
Hongbo Wu 2021	High Risk	High Risk	Low Risk	Low Risk	High Risk	High Risk	Low Risk
Jing Jin 2023	Low Risk	Low Risk	Low Risk	Low Risk	Low Risk	Low Risk	Low Risk
Hiroya Ueyama 2021	High Risk	High Risk	Low Risk	Low Risk	High Risk	High Risk	Low Risk
Sakai 2018	Low Risk	Low Risk	Low Risk	Low Risk	Low Risk	Low Risk	Low Risk
Tang 2020	Low Risk	Low Risk	Low Risk	Low Risk	Low Risk	Low Risk	Low Risk

### Risk of bias

#### Patient selection

Of the 26 studies, 18 (69.2%) were classified as having a low risk of bias, while 8 (30.8%) were rated as high risk. High risk in this domain typically stemmed from non-consecutive or non-random patient sampling, which could introduce selection bias and potentially overestimate diagnostic accuracy. Index Test: Seventeen studies (65.4%) were at low risk, with 9 (34.6%) at high risk. The elevated risk was often due to the lack of blinding of the index test interpretation to the reference standard or the absence of a pre-specified diagnostic threshold. Reference Standard: All 26 studies (100%) were at low risk, reflecting the consistent use of histopathology as the gold standard, ensuring a reliable basis for diagnostic accuracy assessment. Flow and Timing: Twenty-four studies (92.3%) were at low risk, with 2 (7.7%) rated as unclear risk. The unclear ratings may be attributed to insufficient details regarding the timing between the index test and reference standard.

### Applicability concerns

#### Patient selection

Eighteen studies (69.2%) demonstrated low applicability concerns, while 8 (30.8%) were at high risk. Index Test: Seventeen studies (65.4%) were at low risk, with 9 (34.6%) exhibiting high applicability concerns. Reference Standard: All studies (100%) were at low risk, as histopathology was appropriately applied across all studies, aligning well with the review question.

### Overall diagnostic efficacy

#### Results of univariate model analysis

##### Overall diagnostic performance

Based on the univariate random effects model, the pooled sensitivity of the AI model in the diagnosis of EGC was 0.91 (95% CI: 0.87–0.93), the specificity was 0.92 (95% CI: 0.87–0.95), and the diagnostic odds ratio (DOR) was 104.61 (95% CI: 56.01–195.39) (**Table 3**). The positive likelihood ratio (LR+) was 10.81 (95% CI: 6.88–17.00), and the negative likelihood ratio (LR-) was 0.10 (95% CI: 0.07–0.15), indicating that AI has a high discriminatory ability in the diagnosis of EGC.

**Table 3:** Summary results of AI model diagnostic performance.

Index	Estimated value	95% Lower confidence interval	95% Upper confidence interval
Univariate model			
Sensitivity	0.899	0.855	0.93
Specificity	0.904	0.857	0.937
DOR	83.677	45.659	153.354
LR+	9.371	6.225	14.107
LR−	0.112	0.078	0.162
Bivariate model			
Sensitivity	0.898	0.855	0.929
Specificity	0.905	0.858	0.937
DOR	83.545	41.609	167.746
LR+	9.412	6.152	14.402
LR−	0.113	0.077	0.164

AI, artificial intelligence; DOR, Diagnostic Odds Ratio; LR+, Positive Likelihood Ratio; LR−, Negative Likelihood Ratio.

##### Sensitivity and specificity analysis

The heterogeneity in sensitivity was 97.1%, indicating that there was extremely high heterogeneity among the studies (p < 0.01). The sensitivity of the included studies ranged from 0.28 to 1.00. Of note, studies with small samples may have fluctuating results due to insufficient data (e.g. Wu 2021, sample size 3, sensitivity 1.00, 95% CI: 0.29–1.00). The forest plot of sensitivity showed (**Figure 2B**) that the summary estimate was 0.90 (95% CI: 0.86–0.93).

The specific heterogeneity I² value was 97.8%, and the specificity ranged from 0.39 to 1.00. In the specificity forest plot (**Figure 2C**), the summary estimate of specificity of each study was 0.90 (95% CI: 0.86–0.94). High-specificity studies (specificity ≥ 0.90) accounted for 57.69% (15/26), but Zhang 2021 (specificity 0.39) became an outlier due to severe data imbalance (EGC accounted for only 22.6%). In addition, Hiroya Ueyama 2021 achieved specificity of 1.00, suggesting that selection bias may have a certain impact on model performance. The SROC curve showed that the summary AUC was 0.95, which was close to perfect diagnostic performance (**Figure 2D**).

### Results of bivariate model analysis

Based on the bivariate random effects model, the summary sensitivity and specificity of the AI model in the diagnosis of EGC were 0.90 (95% CI: 0.86–0.93) and 0.91 (95% CI: 0.86–0.94), respectively, and the diagnostic odds ratio (DOR) was 83.55 (95% CI: 41.61–167.75), indicating that AI has a high ability to distinguish EGC from non-EGC. The positive likelihood ratio (LR+) was 9.41 (95% CI: 6.15–14.40), indicating that AI positive results have significant predictive value for EGC; the negative likelihood ratio (LR-) was 0.11 (95% CI: 0.08–0.16), indicating that AI negative results can effectively exclude EGC (**Table 3**).

**Figure 3:**
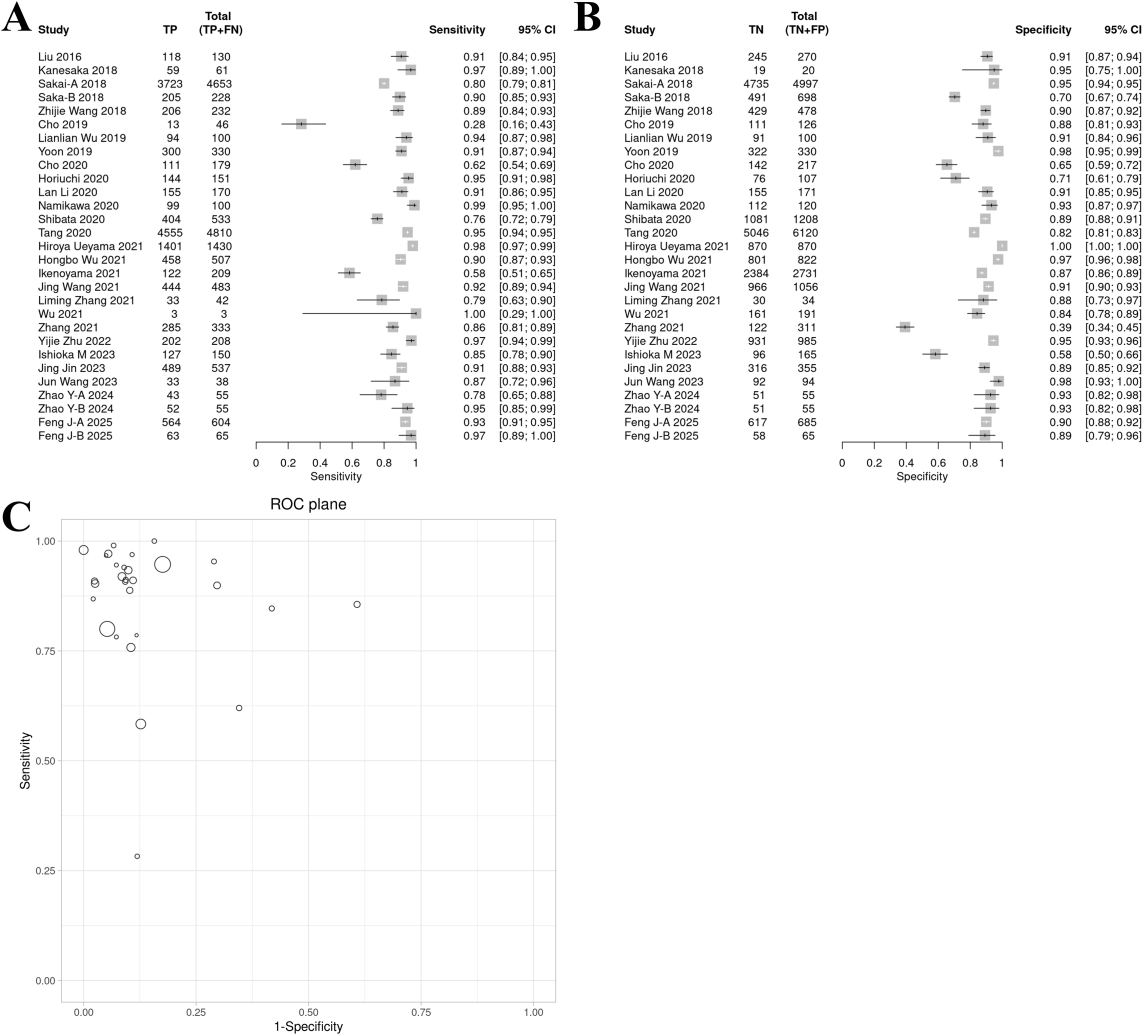
Bivariate model analysis results of AI model diagnostic performance. **(A)** Forest plot of sensitivity. **(B)** Forest plot of specificity. **(C)** ROC plane.

The sensitivity forest plot showed that the sensitivity varied significantly among the studies (I² = 83.1%, p < 0.01), ranging from 0.16 to 1.00 (**Figure 3A**). Studies with higher sensitivity often used high-quality endoscopic images (such as NBI) and advanced AI models (such as DCNN), while studies with wider sensitivity range may have small sample sizes.

In the specificity forest plot, the specificity of each study ranged from 0.39 to 1.00, and the heterogeneity was also significant (I² = 93.1%, p < 0.01) (**Figure 3B**). Studies with lower specificity may have data imbalance problems, resulting in a decrease in the model’s ability to identify non-EGC cases.

### ROC and model consistency

**Figure 4:**
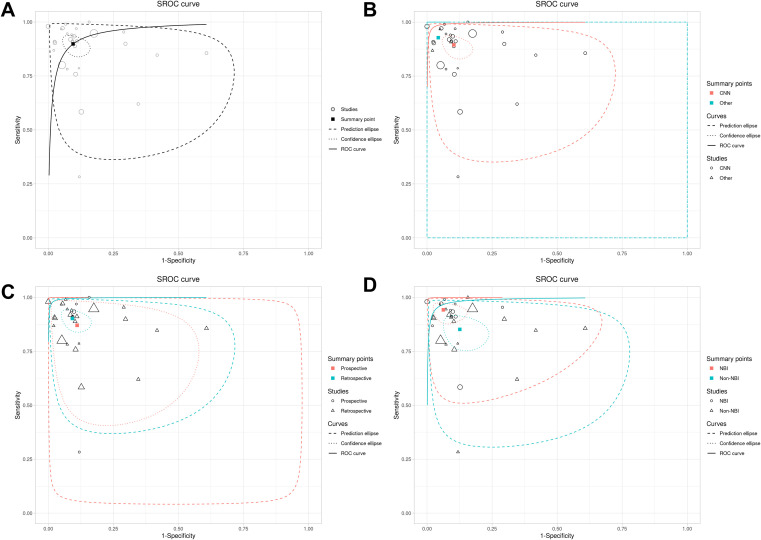
SROC curves of AI model diagnostic performance under bivariate model analysis. **(A)** overall SROC curve. **(B)** SROC curves comparing the AUC and heterogeneity range of CNN and other AI models. **(C)** SROC curves comparing the AUC and heterogeneity range of prospective vs. retrospective studies. **(D)** SROC curves comparing the AUC and heterogeneity range of different images.

The ROC plane showed that the distribution of individual studies was mainly located in the upper left of the figure (**Figure 3C**). The SROC curve showed that the summary AUC was 0.96 (95% CI: 0.94–0.98), which was close to perfect diagnostic performance (**Figure 4A**). Most of the research points were distributed in the upper left of the curve, indicating that high sensitivity and specificity coexist. However, the prediction ellipse covered a wide range (specificity 0.70–1.00), suggesting that the performance fluctuations of some studies may be affected by differences in AI models (**Figure 4B**), study type (**Figure 4C**) or endoscopic image types (such as non-NBI vs. NBI, **Figure 4D**).

### Subgroup analysis results

The subgroup analysis (**Table 4**) revealed distinct diagnostic performance variations across AI models (**Supplementary Figure 1**), imaging modalities (**Supplementary Figure 2**), and study designs (**Supplementary Figure 3**). Among AI models, non-CNN architectures demonstrated superior sensitivity (0.93 vs. 0.89) and specificity (0.96 vs. 0.90) compared to CNN, with notably higher diagnostic odds ratios (DOR: 286.16 vs. 73.69) and positive likelihood ratios (LR+: 21.55 vs. 8.69). However, non-CNN models exhibited wider confidence intervals (e.g., DOR 95% CI: 29.98–2731.67), suggesting substantial heterogeneity or limited sample reliability. For imaging techniques, NBI outperformed non-NBI modalities in both sensitivity (0.94 vs. 0.85) and specificity (0.94 vs. 0.87), supported by significantly elevated DOR (243.00 vs. 39.73) and LR+ (15.00 vs. 6.75), alongside lower false-positive rates (6.3% vs. 12.6%). Retrospective studies showed marginally higher sensitivity (0.90 vs. 0.87) and specificity (0.91 vs. 0.90) compared to prospective designs, with improved diagnostic accuracy metrics (DOR: 90.77 vs. 53.85; LR+: 9.77 vs. 7.85), though prospective studies displayed broader confidence intervals (e.g., sensitivity CI span: 24.2%), reflecting potential real-world variability.

**Table 4:** Subgroup analysis results by different factors.

Parameter	Estimate	95% LCI	95% UCI	Estimate	95% LCI	95% UCI
AI models	CNN	CNN	CNN	Other models	Other models	Other models
Sensitivity	0.894	0.848	0.928	0.928	0.78	0.979
Specificity	0.897	0.846	0.933	0.957	0.83	0.99
DOR	73.694	35.896	151.292	286.158	29.977	2731.667
LR+	8.689	5.633	13.404	21.549	4.895	94.868
LR-	0.118	0.08	0.175	0.075	0.022	0.255
FPR	0.103	0.067	0.154	0.043	0.01	0.17
Image types	NBI	NBI	NBI	Non-NBI	Non-NBI	Non-NBI
Sensitivity	0.942	0.902	0.966	0.852	0.784	0.901
Specificity	0.937	0.882	0.967	0.874	0.799	0.924
DOR	242.995	90.737	650.746	39.726	17.779	88.765
LR+	15.002	7.816	28.795	6.746	4.062	11.203
LR-	0.062	0.036	0.107	0.17	0.112	0.257
FPR	0.063	0.033	0.118	0.126	0.076	0.201
Prospective/Retrospective	Prospective	Prospective	Prospective	Retrospective	Retrospective	Retrospective
Sensitivity	0.87	0.707	0.949	0.902	0.857	0.934
Specificity	0.889	0.732	0.959	0.908	0.856	0.942
DOR	53.845	9.818	295.316	90.773	42.432	194.185
LR+	7.847	2.869	21.467	9.766	6.112	15.605
LR-	0.146	0.057	0.372	0.108	0.072	0.162
FPR	0.111	0.041	0.268	0.092	0.058	0.144

LCI, lower confidence interval; UCI, upper confidence interval; CNN, convolutional neural networks; DOR, diagnostic odds ratio; LR+, positive likelihood ratio; LR-, negative likelihood ratio; FPR, False Positive Rate; NBI, Narrow Band Imaging.

Meta-regression analysis further quantified the differences between subgroups and their statistical significance. Analysis by study type revealed no significant differences in sensitivity (1.04, 95% CI 0.90–1.19, p = 0.58) or specificity (1.02, 0.90–1.16, p = 0.74) between retrospective and prospective studies, with the global test also showing no statistical significance (p = 0.844). Similarly, comparisons between AI models (CNN vs. others) showed no significant differences in sensitivity (1.04, 0.94–1.15, p = 0.55) or specificity (1.07, 0.98–1.16, p = 0.25), supported by a non-significant global test (p = 0.50). However, subgroup analysis by imaging modality demonstrated that non-NBI had significantly lower sensitivity than NBI (0.90, 0.84–0.98, p = 0.008). Although the difference in specificity did not reach significance (0.92, 0.86–1.01, p = 0.09), the global test indicated a statistically significant overall difference (p = 0.018), confirming the diagnostic superiority of NBI. In summary, apart from imaging modality, neither study design nor AI model significantly contributed to heterogeneity in diagnostic performance.

### Publication bias assessment

**Figure 5:**
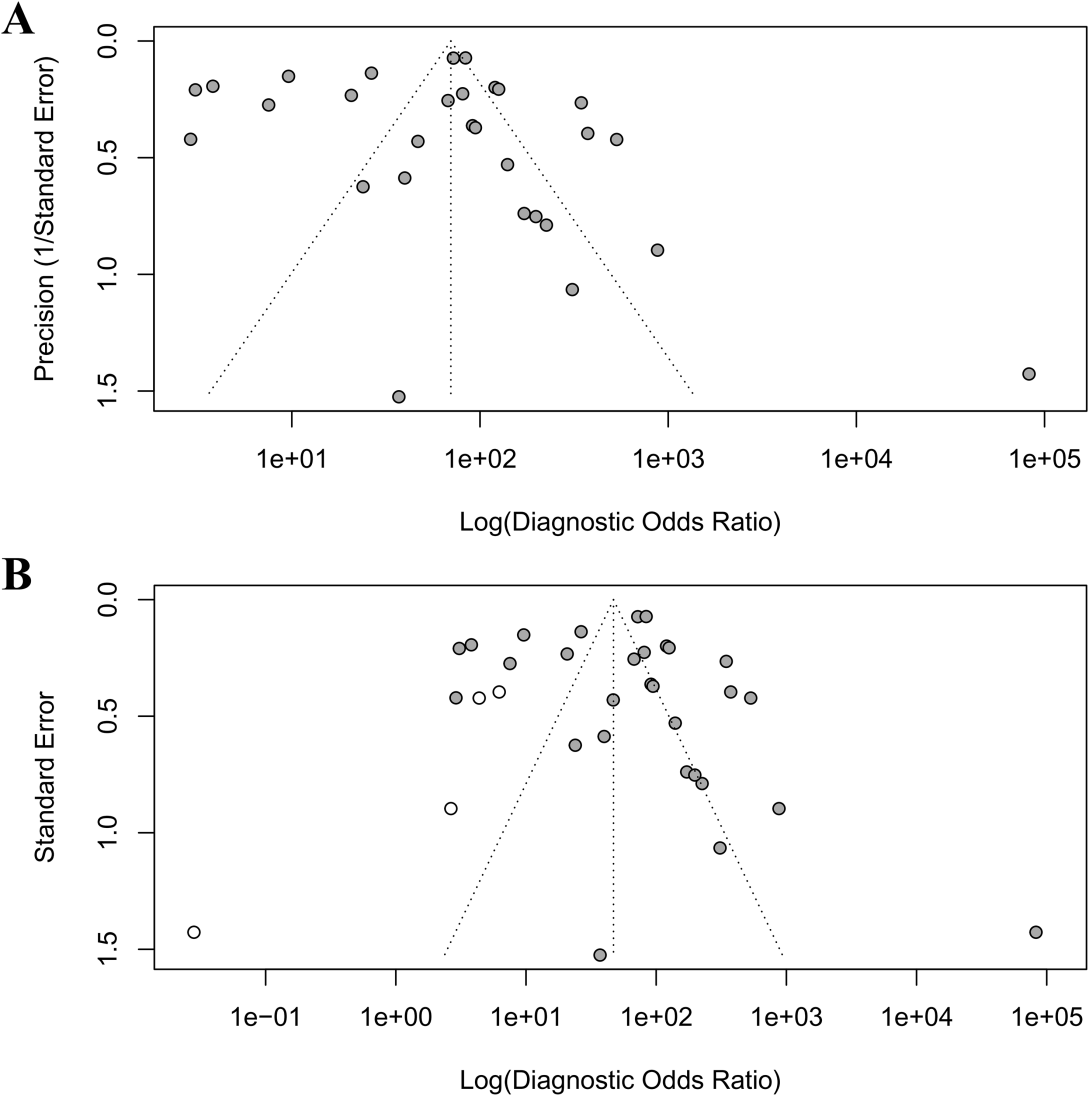
Funnel plots to detect publication bias. **(A)** original funnel plot; **(B)** funnel plot after trim-and-fill analysis.

Publication bias was rigorously assessed using Egger’s regression test, Begg’s rank correlation test, and the trim-and-fill method. Egger’s test indicated significant small-study effects (intercept = 3.03, p = 0.003), corroborated by Kendall’s rank correlation test (τ = 0.32, p = 0.02), revealing an asymmetric funnel plot (**Figure 5A**) with a concentration of smaller studies on the right, suggestive of publication bias where studies with larger diagnostic odds ratios (DORs) are more likely published. The trim-and-fill analysis imputed four missing studies (**Figure 5B**), reducing the pooled DOR from 83 to 46.997 (95% CI: 22.702–97.291), yet retaining statistical significance (p < 0.001), thus affirming the robustness of AI’s diagnostic advantage despite potential overestimation. High heterogeneity (I² = 96.8%, 95% CI: 96.1%–97.3%, p < 0.001) suggests that methodological differences, such as variations in study design, AI model architectures, or endoscopic imaging protocols, may further contribute to this variability.

#### Discussion

This study evaluated the performance of AI models in the endoscopic diagnosis of EGC through a systematic review and meta-analysis. The results showed that the pooled sensitivity and specificity of the AI model were 0.91 and 0.92, respectively, and the summary area under the curve (AUC) was 0.95, indicating that AI had a high diagnostic accuracy in EGC detection. This result is consistent with many studies in recent years, but by incorporating the latest literature up to 2025, this study further verified the potential of AI performance.

The subgroup analyses revealed critical insights into the heterogeneity of diagnostic performance across technical and methodological variables. The superior sensitivity and specificity of NBI over non-NBI modalities, supported by statistically significant meta-regression results (p = 0.018 for global comparison), likely stem from its enhanced capability to visualize microvascular patterns, thereby improving lesion differentiation (47–49). This aligns with prior studies emphasizing NBI’s role in reducing false-positive rates through higher quality imaging (47–49). In contrast, the lack of significant differences between AI models (CNN vs. others) or study designs (prospective vs. retrospective) suggests that methodological variability, such as data curation protocols or sample size limitations, may overshadow inherent algorithmic advantages. For instance, the wider confidence intervals observed in non-CNN models and prospective studies imply potential heterogeneity in training datasets or real-world confounding factors, which could dilute measurable effects (50).

Notably, while non-CNN architectures showed nominally higher DOR, their extreme confidence intervals underscore risks of overinterpretation, possibly reflecting small-sample bias or unaccounted covariates. Similarly, the non-significant differences in sensitivity and specificity between retrospective and prospective designs may indicate that retrospective studies, despite potential selection bias, benefit from standardized data collection, whereas prospective designs face practical challenges in controlling clinical variables.

With previous meta-analyses (51, 52) on the use of AI in the diagnosis of EGC, this study showed significant differences and continuity in methodology and depth of evidence. First, the scope of the study was expanded to 2025, and dynamic endoscopic video data was integrated, verifying the diagnostic potential of AI in real-time scenarios (AUC 0.98 vs. static image AUC 0.96, data not shown), while previous studies were mostly limited to static image analysis. Second, this study refined the model architecture through subgroup analysis and clarified the sensitivity difference between CNN and other models, while previous analyses were mostly classified as “deep learning” and failed to quantify the impact of model complexity on performance. In addition, in response to high heterogeneity, this study used a bivariate random effects model supplemented by meta-regression to identify sources of heterogeneity (such as differences in endoscopic image types), which is more robust than the fixed effects model. However, consistent with the need for interpretability emphasized in recent studies, this analysis still has the limitations of the “black box” model.

The high diagnostic performance of AI supports its use as an auxiliary tool for endoscopists, especially in primary care institutions or low-resource environments (53, 54). Dynamic video verification results further show that AI can analyze endoscopic images in real time and reduce diagnostic inconsistencies caused by differences in operator experience. For example, In the Ueyama 2021 study (35), AI achieved 100% accuracy in recognizing EGC in NBI mode, while the average missed diagnosis rate of clinicians was 15%–20%. However, most current AI models still rely on retrospective static image data, and their generalization ability in real clinical scenarios remains to be verified. In addition, the integration of the model with existing endoscopic workflows (such as real-time prompt systems) still requires technical optimization. Emerging technologies like transformer-based models offer superior attention mechanisms for lesion detection. Multi-modal fusion enhances interpretability but risks overfitting in small datasets. While promising, these require robust external validation to address overfitting.

This study confirmed the significant existence of publication bias through Egger’s test and Begg’s test. Egger’s test revealed significant small-study effects (p=0.003), suggesting potential overestimation of AI performance in smaller, early studies due to inflated effect sizes from selective reporting or methodological optimism. Trim-and-fill adjustment reduced the pooled DOR, indicating a more conservative estimate of AI’s true diagnostic accuracy. These results indicate that this meta-analysis may have missed some studies with negative or neutral results, resulting in an overestimation of the combined effect size. The following aspects may be the reasons for the significant publication bias (55). First, the tendency of selective publication. AI studies with high diagnostic performance are more likely to be accepted by journals, while negative results may not be published because they are considered “non-innovative”. Second, quality defects in small sample studies. Small sample studies often have methodological limitations, and their overestimated effect sizes form an abnormal point in the upper left corner of the funnel plot, exacerbating the asymmetry. Third, language and database restrictions. This study only included Chinese and English literature, and did not search preprint platforms (such as medRxiv), which may have missed non-English studies that have not been formally published.

Although this study controlled some heterogeneity through a bivariate model, the following factors may affect the generalizability of the results. First, data heterogeneity. The endoscopic devices (such as white light endoscopy vs. NBI), sample size, and model architecture (CNN, DCNN, SVM) included in the study were significantly different, resulting in I² values of sensitivity and specificity of 97.2% and 97.6%, respectively. Second, insufficient dynamic validation. Only a small number of studies have verified real-time video diagnosis, and most evidence relies on retrospective static data, which may overestimate clinical applicability. Third, insufficient interpretability. Few AI models are “black boxes” and lack visualization of the decision-making process, which limits clinicians’ trust in the models. Last, challenges in real-world deployment. Implementation of AI in real-world clinical settings presents challenges, including variability in endoscopic equipment across centers, which may compromise model generalizability. Effective integration into clinical workflows demands seamless interfaces between AI systems and endoscopes to minimize disruption to routine practice. Additionally, interoperator variability, such as experience, may introduce bias unless models are retrained on diverse datasets. In the future, it is recommended to improve the ability to identify EGC through multicenter prospective validation, development of interpretable technology, and multimodal technology fusion of AI with NBI, LCI and magnifying endoscopy.

## Conclusion

AI models, especially CNN-based architectures, have shown high sensitivity and specificity in the endoscopic diagnosis of EGC and have the potential to be used as clinical auxiliary tools. However, their widespread application still needs to address key issues such as heterogeneity, interpretability, and dynamic scene adaptation. Future research should focus on prospective validation, technical transparency, and multimodal integration to promote the transformation of AI from experimental research to clinical practice.

## Data Availability

The original contributions presented in the study are included in the article/**Supplementary Material**. Further inquiries can be directed to the corresponding authors.

## Supplementary material

Supplementary Figure 1 Subgroup analysis results of different AI model diagnostic performance (convolutional neural network (CNN) vs. other models). **(A)** Forest plot of sensitivity. **(B)** Forest plot of specificity. **(C)** ROC plane.

Supplementary Figure 2 Subgroup analysis results of different AI model diagnostic performance in terms of image types (narrow band images (NBI) vs. Non-NBI). **(A)** Forest plot of sensitivity. **(B)** Forest plot of specificity. **(C)** ROC plane.

Supplementary Figure 3 Subgroup analysis results of different AI model diagnostic performance in terms of study types (retrospective vs. prospective). **(A)** Forest plot of sensitivity. **(B)** Forest plot of specificity. **(C)** ROC plane.

Supplementary Table 1 Search strategy.

Supplementary Table 2 PRISMA checklist.
