# Supplementary figures and images for: Comparative diagnostic accuracy of different artificial intelligence models for early gastric cancer: a systematic review and meta-analysis
Source: Front Oncol. 2025 Nov 18;15:1670843. doi: 10.3389/fonc.2025.1670843 (PMC12668911; doi:10.3389/fonc.2025.1670843)

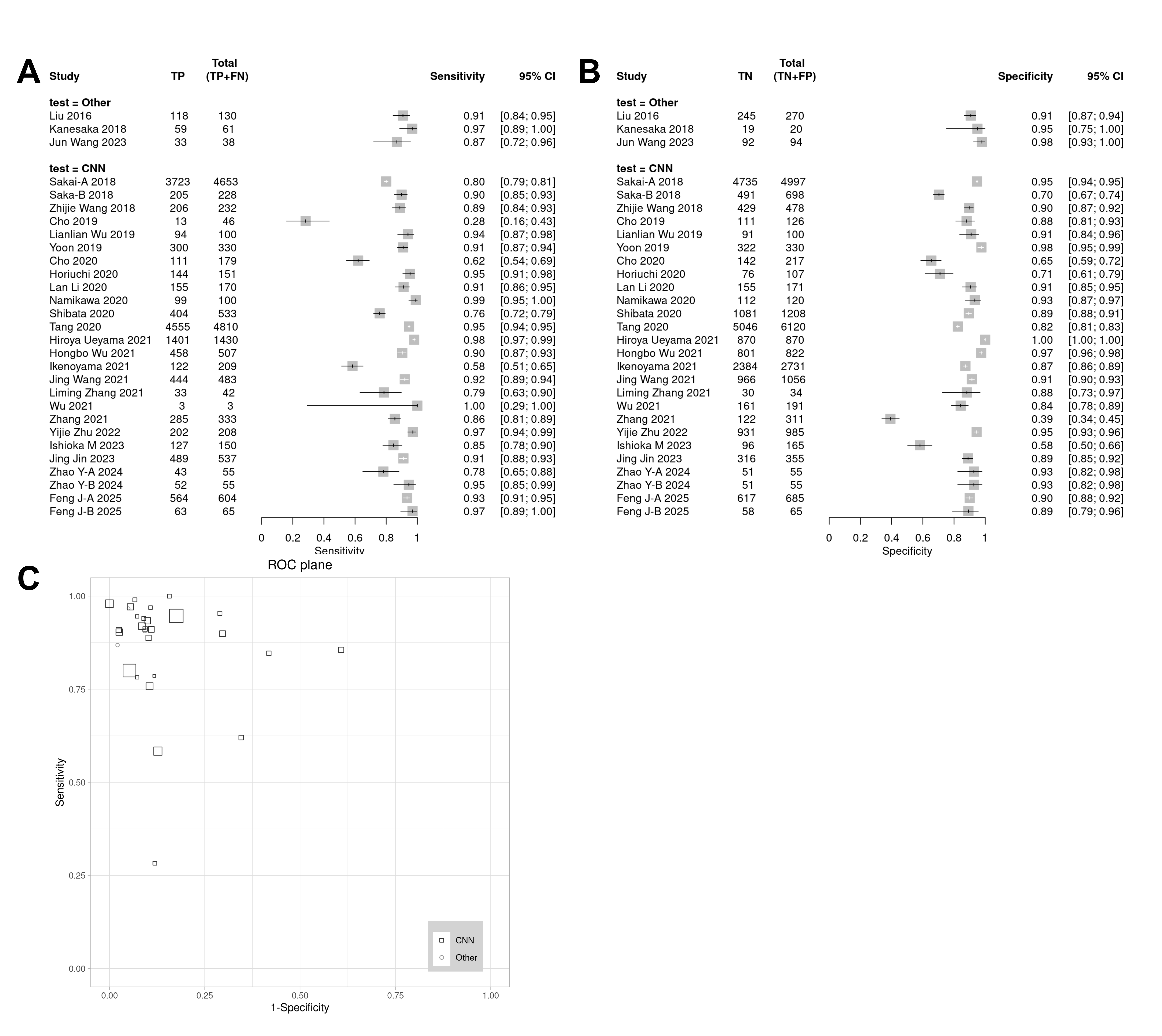

Supplement: Supplementary Figure 1 — Subgroup analysis results of different AI model diagnostic performance (convolutional neural network (CNN) vs. other models). (A) Forest plot of sensitivity. (B) Forest plot of specificity. (C) ROC plane. [file Image1.tif]

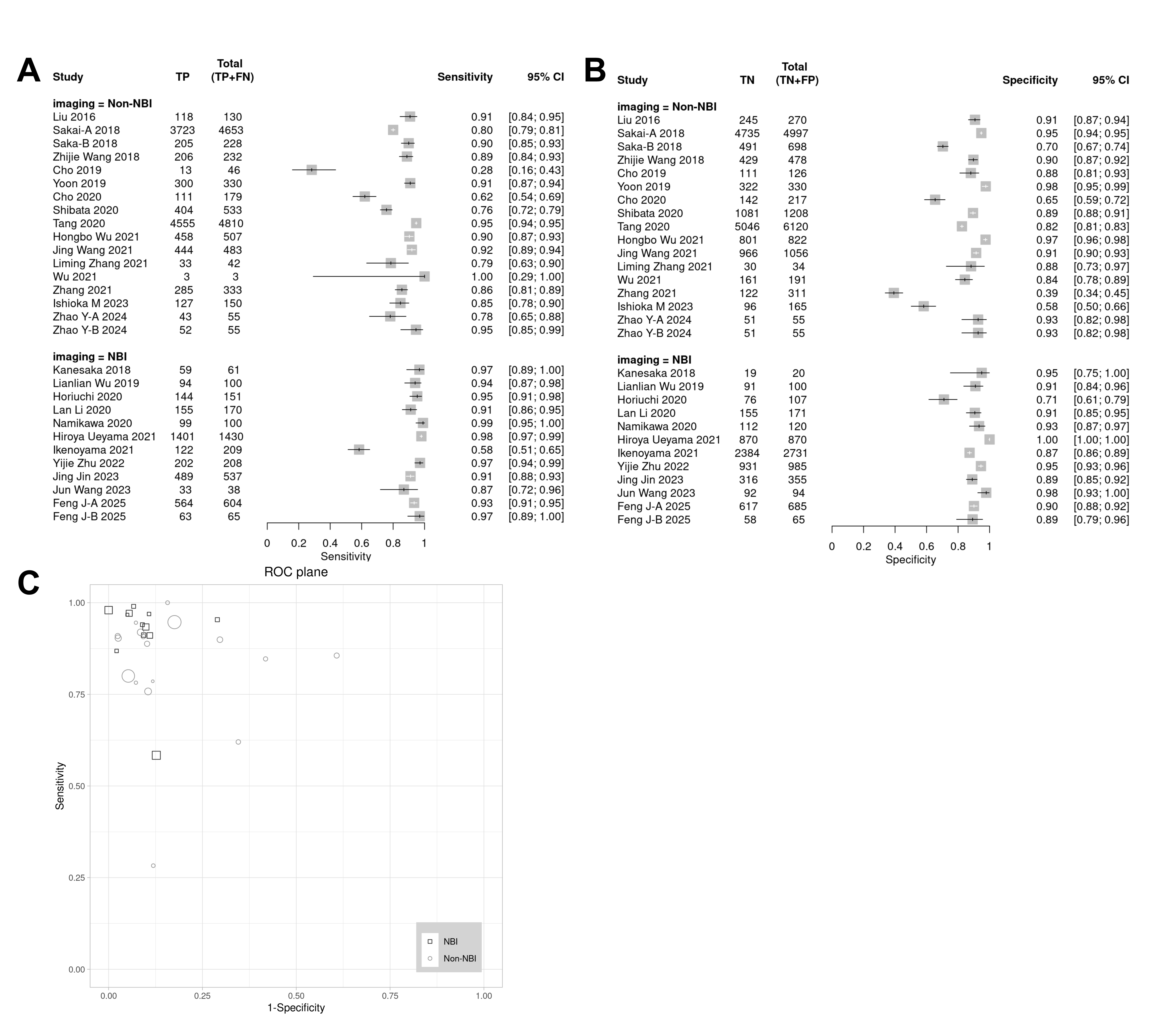

Supplement: Supplementary Figure 2 — Subgroup analysis results of different AI model diagnostic performance in terms of image types (narrow band images (NBI) vs. Non-NBI). (A) Forest plot of sensitivity. (B) Forest plot of specificity. (C) ROC plane. [file Image2.tif]

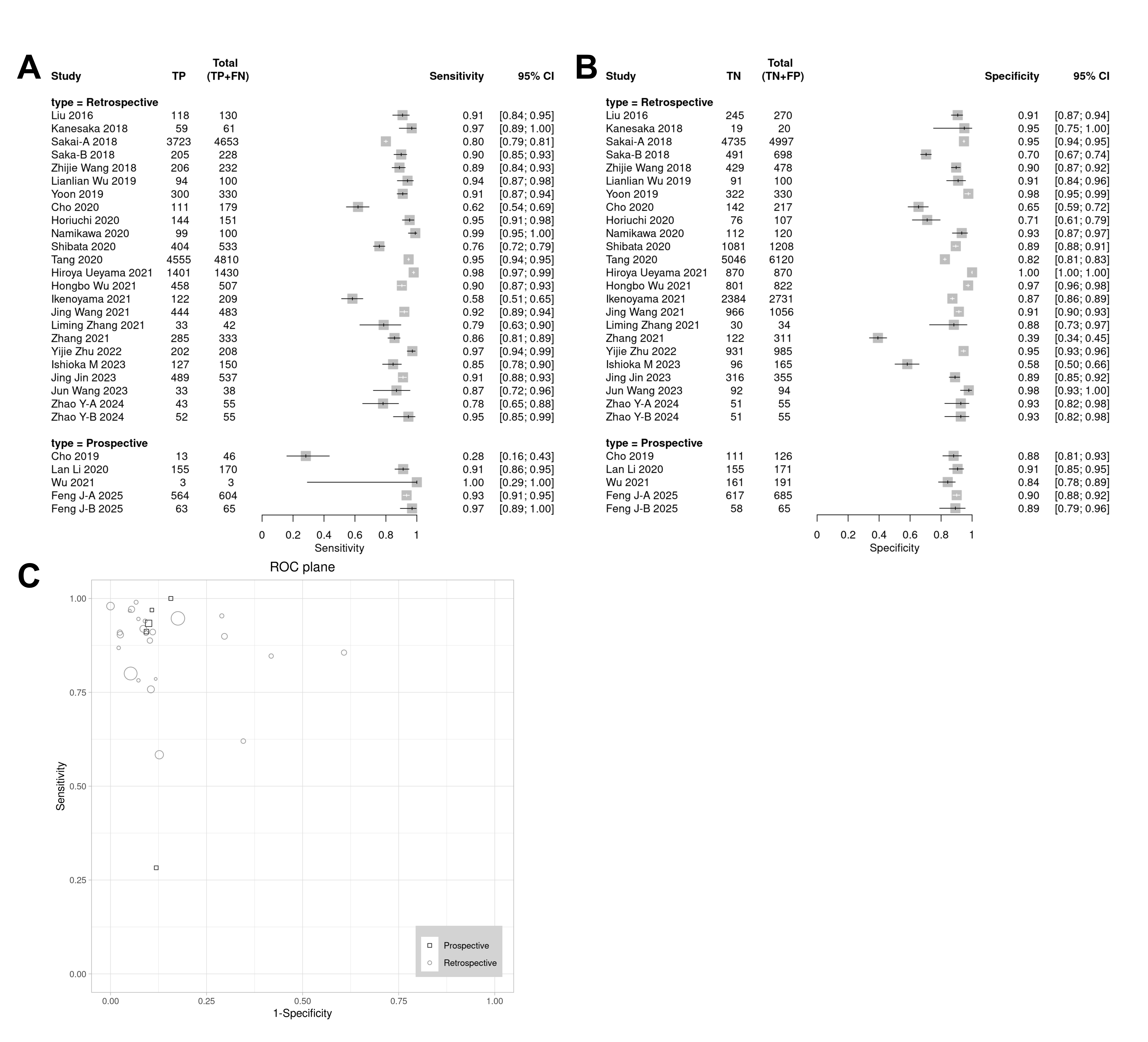

Supplement: Supplementary Figure 3 — Subgroup analysis results of different AI model diagnostic performance in terms of study types (retrospective vs. prospective). (A) Forest plot of sensitivity. (B) Forest plot of specificity. (C) ROC plane. [file Image3.tif]
