# Supplementary material for: Comparative diagnostic accuracy of different artificial intelligence models for early gastric cancer: a systematic review and meta-analysis
Source: Front Oncol. 2025 Nov 18;15:1670843. doi: 10.3389/fonc.2025.1670843 (PMC12668911; doi:10.3389/fonc.2025.1670843)
Supplement: Supplementary Table 1 — Search strategy. [file DataSheet1.docx]

Pubmed

| Search number | Query | Sort By | Filters | Search Details |
| --- | --- | --- | --- | --- |
| 9 | #8 NOT #5 | Publication Date | | (("Stomach Neoplasms"[MeSH Terms] OR "early gastric cancer"[Title/Abstract] OR "early gastric carcinoma"[Title/Abstract] OR "EGC"[Title/Abstract] OR ("gastric"[Title/Abstract] AND ("cancer"[Title/Abstract] OR "carcinoma"[Title/Abstract] OR "neoplasm*"[Title/Abstract]) AND "early"[Title/Abstract])) AND ("Artificial Intelligence"[MeSH Terms] OR "Machine Learning"[MeSH Terms] OR "Deep Learning"[MeSH Terms] OR "Algorithms"[MeSH Terms] OR "AI"[Title/Abstract] OR "CNN"[Title/Abstract] OR "neural network*"[Title/Abstract] OR "support vector machine*"[Title/Abstract] OR "random forest*"[Title/Abstract]) AND ("Endoscopy"[MeSH Terms] OR "Endoscopic"[Title/Abstract] OR "Gastroscopy"[Title/Abstract] OR "Endoscopic Imaging"[Title/Abstract]) AND ("Sensitivity and Specificity"[MeSH Terms] OR "ROC Curve"[MeSH Terms] OR "AUC"[Title/Abstract] OR "predictive value"[Title/Abstract] OR "diagnostic odds ratio"[Title/Abstract])) NOT ("Animals"[MeSH Terms] NOT "Humans"[MeSH Terms]) |
| 8 | #7 AND #2 AND #3 AND #4 | Publication Date | | ("Stomach Neoplasms"[MeSH Terms] OR "early gastric cancer"[Title/Abstract] OR "early gastric carcinoma"[Title/Abstract] OR "EGC"[Title/Abstract] OR ("gastric"[Title/Abstract] AND ("cancer"[Title/Abstract] OR "carcinoma"[Title/Abstract] OR "neoplasm*"[Title/Abstract]) AND "early"[Title/Abstract])) AND ("Artificial Intelligence"[MeSH Terms] OR "Machine Learning"[MeSH Terms] OR "Deep Learning"[MeSH Terms] OR "Algorithms"[MeSH Terms] OR "AI"[Title/Abstract] OR "CNN"[Title/Abstract] OR "neural network*"[Title/Abstract] OR "support vector machine*"[Title/Abstract] OR "random forest*"[Title/Abstract]) AND ("Endoscopy"[MeSH Terms] OR "Endoscopic"[Title/Abstract] OR "Gastroscopy"[Title/Abstract] OR "Endoscopic Imaging"[Title/Abstract]) AND ("Sensitivity and Specificity"[MeSH Terms] OR "ROC Curve"[MeSH Terms] OR "AUC"[Title/Abstract] OR "predictive value"[Title/Abstract] OR "diagnostic odds ratio"[Title/Abstract]) |
| 7 | ("Stomach Neoplasms"[Mesh]) OR "early gastric cancer"[tiab] OR "early gastric carcinoma"[tiab] OR "EGC"[tiab] OR ("gastric"[tiab] AND (cancer[tiab] OR carcinoma[tiab] OR neoplasm*[tiab]) AND early[tiab]) ) | Publication Date | | "Stomach Neoplasms"[MeSH Terms] OR "early gastric cancer"[Title/Abstract] OR "early gastric carcinoma"[Title/Abstract] OR "EGC"[Title/Abstract] OR ("gastric"[Title/Abstract] AND ("cancer"[Title/Abstract] OR "carcinoma"[Title/Abstract] OR "neoplasm*"[Title/Abstract]) AND "early"[Title/Abstract]) |
| 6 | #1 AND #2 AND #3 AND #4 | Publication Date | | (("Stomach Neoplasms"[MeSH Terms] AND "Early Diagnosis"[MeSH Terms]) OR "early gastric cancer"[Title/Abstract] OR "early gastric carcinoma"[Title/Abstract] OR "EGC"[Title/Abstract] OR ("gastric"[Title/Abstract] AND ("cancer"[Title/Abstract] OR "carcinoma"[Title/Abstract] OR "neoplasm*"[Title/Abstract]) AND "early"[Title/Abstract])) AND ("Artificial Intelligence"[MeSH Terms] OR "Machine Learning"[MeSH Terms] OR "Deep Learning"[MeSH Terms] OR "Algorithms"[MeSH Terms] OR "AI"[Title/Abstract] OR "CNN"[Title/Abstract] OR "neural network*"[Title/Abstract] OR "support vector machine*"[Title/Abstract] OR "random forest*"[Title/Abstract]) AND ("Endoscopy"[MeSH Terms] OR "Endoscopic"[Title/Abstract] OR "Gastroscopy"[Title/Abstract] OR "Endoscopic Imaging"[Title/Abstract]) AND ("Sensitivity and Specificity"[MeSH Terms] OR "ROC Curve"[MeSH Terms] OR "AUC"[Title/Abstract] OR "predictive value"[Title/Abstract] OR "diagnostic odds ratio"[Title/Abstract]) |
| 5 | "Animals"[Mesh] NOT "Humans"[Mesh] | Publication Date | | "Animals"[MeSH Terms] NOT "Humans"[MeSH Terms] |
| 4 | "Diagnostic Accuracy"[Mesh] OR "Sensitivity and Specificity"[Mesh] OR "ROC Curve"[Mesh] OR "AUC"[tiab] OR "predictive value"[tiab] OR "diagnostic odds ratio"[tiab] | Publication Date | | "Sensitivity and Specificity"[MeSH Terms] OR "ROC Curve"[MeSH Terms] OR "AUC"[Title/Abstract] OR "predictive value"[Title/Abstract] OR "diagnostic odds ratio"[Title/Abstract] |
| 3 | "Endoscopy"[Mesh] OR "Endoscopic"[tiab] OR "Gastroscopy"[tiab] OR "Endoscopic Imaging"[tiab] | Publication Date | | "Endoscopy"[MeSH Terms] OR "Endoscopic"[Title/Abstract] OR "Gastroscopy"[Title/Abstract] OR "Endoscopic Imaging"[Title/Abstract] |
| 2 | "Artificial Intelligence"[Mesh] OR "Machine Learning"[Mesh] OR "Deep Learning"[Mesh] OR "Algorithms"[Mesh] OR "AI"[tiab] OR "CNN"[tiab] OR "neural network*"[tiab] OR "support vector machine*"[tiab] OR "random forest*"[tiab] | Publication Date | | "Artificial Intelligence"[MeSH Terms] OR "Machine Learning"[MeSH Terms] OR "Deep Learning"[MeSH Terms] OR "Algorithms"[MeSH Terms] OR "AI"[Title/Abstract] OR "CNN"[Title/Abstract] OR "neural network*"[Title/Abstract] OR "support vector machine*"[Title/Abstract] OR "random forest*"[Title/Abstract] |
| 1 | ("Stomach Neoplasms"[Mesh] AND "Early Diagnosis"[Mesh]) OR "early gastric cancer"[tiab] OR "early gastric carcinoma"[tiab] OR "EGC"[tiab] OR ("gastric"[tiab] AND (cancer[tiab] OR carcinoma[tiab] OR neoplasm*[tiab]) AND early[tiab]) ) | Publication Date | | ("Stomach Neoplasms"[MeSH Terms] AND "Early Diagnosis"[MeSH Terms]) OR "early gastric cancer"[Title/Abstract] OR "early gastric carcinoma"[Title/Abstract] OR "EGC"[Title/Abstract] OR ("gastric"[Title/Abstract] AND ("cancer"[Title/Abstract] OR "carcinoma"[Title/Abstract] OR "neoplasm*"[Title/Abstract]) AND "early"[Title/Abstract]) |

Embase

| No. | Query |
| --- | --- |
| #5 | ('early gastric cancer'/exp OR 'early gastric cancer' OR 'egc' OR (gastric:ti,ab,kw AND cancer:ti,ab,kw AND early:ti,ab,kw)) AND ('machine learning'/exp OR 'deep learning'/exp OR 'neural network'/exp OR 'random forest'/exp OR ai:ti,ab,kw OR cnn:ti,ab,kw OR 'support vector machine':ti,ab,kw) AND ('diagnostic accuracy'/exp OR 'sensitivity and specificity'/exp OR 'roc curve'/exp OR auc:ti,ab,kw OR 'predictive value':ti,ab,kw OR 'diagnostic odds ratio':ti,ab,kw) AND ([article]/lim OR [article in press]/lim) AND [humans]/lim AND [clinical study]/lim |
| #4 | ('early gastric cancer'/exp OR 'early gastric cancer' OR 'egc' OR (gastric:ti,ab,kw AND cancer:ti,ab,kw AND early:ti,ab,kw)) AND ('machine learning'/exp OR 'deep learning'/exp OR 'neural network'/exp OR 'random forest'/exp OR ai:ti,ab,kw OR cnn:ti,ab,kw OR 'support vector machine':ti,ab,kw) AND ('diagnostic accuracy'/exp OR 'sensitivity and specificity'/exp OR 'roc curve'/exp OR auc:ti,ab,kw OR 'predictive value':ti,ab,kw OR 'diagnostic odds ratio':ti,ab,kw) |
| #3 | 'diagnostic accuracy'/exp OR 'sensitivity and specificity'/exp OR 'roc curve'/exp OR auc:ti,ab,kw OR 'predictive value':ti,ab,kw OR 'diagnostic odds ratio':ti,ab,kw |
| #2 | 'machine learning'/exp OR 'deep learning'/exp OR 'neural network'/exp OR 'random forest'/exp OR ai:ti,ab,kw OR cnn:ti,ab,kw OR 'support vector machine':ti,ab,kw |
| #1 | 'early gastric cancer'/exp OR 'early gastric cancer' OR 'egc' OR (gastric:ti,ab,kw AND cancer:ti,ab,kw AND early:ti,ab,kw) |

Cochrane

ID Search Hits

#1 (Stomach Neoplasms):ti,ab,kw OR ("gastric cancer"):ti,ab,kw OR (EGC):ti,ab,kw OR ("gastric carcinoma"):ti,ab,kw (Word variations have been searched) 10133

#2 ("artificial intelligence"):ti,ab,kw OR ("machine learning"):ti,ab,kw OR (Deep Learning):ti,ab,kw OR (AI):ti,ab,kw AND (CNN):ti,ab,kw (Word variations have been searched) 6772

#3 (neural network):ti,ab,kw OR ("support-vector machine"):ti,ab,kw OR (random forest):ti,ab,kw (Word variations have been searched) 5517

#4 ("endoscopy"):ti,ab,kw OR (endoscopic):ti,ab,kw OR (gastroscopy):ti,ab,kw OR (endoscopic imaging):ti,ab,kw (Word variations have been searched) 36630

#5 (Diagnostic Accuracy):ti,ab,kw OR ("sensitivity"):ti,ab,kw OR ("specificity"):ti,ab,kw OR ("predictive value positive"):ti,ab,kw OR (ROC Curve):ti,ab,kw (Word variations have been searched) 256896

#6 #1 AND (#2 OR #3) AND #4 AND #5 19

CNKI search stragety

(SU='人工智能' OR KY='人工智能' OR TI='人工智能' OR AB='人工智能' OR SU='机器学习' OR KY='机器学习' OR TI='机器学习' OR AB='机器学习' OR SU='深度学习' OR KY='深度学习' OR TI='深度学习' OR AB='深度学习' OR SU='卷积神经网络' OR KY='卷积神经网络' OR TI='卷积神经网络' OR AB='卷积神经网络' OR SU='支持向量机' OR KY='支持向量机' OR TI='支持向量机' OR AB='支持向量机' OR SU='随机森林' OR KY='随机森林' OR TI='随机森林' OR AB='随机森林' OR SU='artificial intelligence' OR KY='artificial intelligence' OR TI='artificial intelligence' OR AB='artificial intelligence' OR SU='machine learning' OR KY='machine learning' OR TI='machine learning' OR AB='machine learning' OR SU='deep learning' OR KY='deep learning' OR TI='deep learning' OR AB='deep learning' OR SU='convolutional neural network' OR KY='convolutional neural network' OR TI='convolutional neural network' OR AB='convolutional neural network' OR SU='support vector machine' OR KY='support vector machine' OR TI='support vector machine' OR AB='support vector machine' OR SU='random forest' OR KY='random forest' OR TI='random forest' OR AB='random forest')

AND

(SU='早期胃癌' OR KY='早期胃癌' OR TI='早期胃癌' OR AB='早期胃癌' OR SU='早期胃肿瘤' OR KY='早期胃肿瘤' OR TI='早期胃肿瘤' OR AB='早期胃肿瘤' OR SU='early gastric cancer' OR KY='early gastric cancer' OR TI='early gastric cancer' OR AB='early gastric cancer' OR SU='early stomach neoplasm' OR KY='early stomach neoplasm' OR TI='early stomach neoplasm' OR AB='early stomach neoplasm')

AND

(SU='内镜' OR KY='内镜' OR TI='内镜' OR AB='内镜' OR SU='胃镜' OR KY='胃镜' OR TI='胃镜' OR AB='胃镜' OR SU='endoscopy' OR KY='endoscopy' OR TI='endoscopy' OR AB='endoscopy' OR SU='gastroscopy' OR KY='gastroscopy' OR TI='gastroscopy' OR AB='gastroscopy')

AND

(SU='诊断' OR KY='诊断' OR TI='诊断' OR AB='诊断' OR SU='检测' OR KY='检测' OR TI='检测' OR AB='检测' OR SU='diagnosis' OR KY='diagnosis' OR TI='diagnosis' OR AB='diagnosis' OR SU='detection' OR KY='detection' OR TI='detection' OR AB='detection')

AND

(SU='准确性' OR KY='准确性' OR TI='准确性' OR AB='准确性' OR SU='敏感性' OR KY='敏感性' OR TI='敏感性' OR AB='敏感性' OR SU='特异性' OR KY='特异性' OR TI='特异性' OR AB='特异性' OR SU='accuracy' OR KY='accuracy' OR TI='accuracy' OR AB='accuracy' OR SU='sensitivity' OR KY='sensitivity' OR TI='sensitivity' OR AB='sensitivity' OR SU='specificity' OR KY='specificity' OR TI='specificity' OR AB='specificity')

Web of Science search stragety

(TS=("artificial intelligence" OR "machine learning" OR "deep learning" OR "convolutional neural network" OR "support vector machine" OR "random forest" OR "AI" OR "ML" OR "DL" OR "CNN" OR "SVM" OR "RF"))

AND

(TS=("early gastric cancer" OR "early stomach neoplasm" OR "early gastric carcinoma" OR "EGC" OR "ESN"))

AND

(TS=("endoscopy" OR "gastroscopy" OR "endoscopic examination" OR "endoscopic diagnosis" OR "GI endoscopy"))

AND

(TS=("diagnosis" OR "detection" OR "classification" OR "screening" OR "prediction"))

AND

(TS=("accuracy" OR "sensitivity" OR "specificity" OR "AUC" OR "precision" OR "recall" OR "validation"))
